# Constructing a Novel Amino Acid Metabolism Signature: A New Perspective on Pheochromocytoma Diagnosis, Immune Landscape, and Immunotherapy

**DOI:** 10.1007/s10528-024-10733-5

**Published:** 2024-03-25

**Authors:** Zechen Yan, Yongkun Luan, Yu Wang, Yilin Ren, Zhiyuan Li, Luyang Zhao, Linnuo Shen, Xiaojie Yang, Tonghu Liu, Yukui Gao, Weibo Sun

**Affiliations:** 1 BGI College and Henan Institute of Medical and Pharmaceutical Sciences, Zhengzhou University, Zhengzhou, 450001 Henan People’s Republic of China; 2 Department of Surgery, The First Affiliated Hospital of Zhengzhou University, Zhengzhou, 450001 Henan People’s Republic of China; 3 Henan Engineering Research Center of Tumor Molecular Diagnosis and Treatment, Zhengzhou, 450001 Henan People’s Republic of China; 4 Institute of Molecular Cancer Surgery, Zhengzhou University, Zhengzhou, 450001 Henan People’s Republic of China; 5 Department of Radiation Oncology and Oncology, Henan Provincial People’s Hospital, Zhengzhou University People’s Hospital, Henan University People’s Hospital, Zhengzhou, 450000 China

**Keywords:** Pheochromocytoma/paraganglioma, Amino acid metabolism, Diagnosis, Risk prediction, Immune microenvironment, Immunotherapy

## Abstract

Pheochromocytoma/paraganglioma (PGPG) is a rare neuroendocrine tumor. Amino acid metabolism is crucial for energy production, redox balance, and metabolic pathways in tumor cell proliferation. This study aimed to build a risk model using amino acid metabolism-related genes, enhancing PGPG diagnosis and treatment decisions. We analyzed RNA-sequencing data from the PCPG cohort in the GEO dataset as our training set and validated our findings using the TCGA dataset and an additional clinical cohort. WGCNA and LASSO were utilized to identify hub genes and develop risk prediction models. The single-sample gene set enrichment analysis, MCPCOUNTER, and ESTIMATE algorithm calculated the relationship between amino acid metabolism and immune cell infiltration in PCPG. The TIDE algorithm predicted the immunotherapy efficacy for PCPG patients. The analysis identified 292 genes with differential expression, which are involved in amino acid metabolism and immune pathways. Six genes (DDC, SYT11, GCLM, PSMB7, TYRO3, AGMAT) were identified as crucial for the risk prediction model. Patients with a high-risk profile demonstrated reduced immune infiltration but potentially higher benefits from immunotherapy. Notably, DDC and SYT11 showed strong diagnostic and prognostic potential. Validation through quantitative Real-Time Polymerase Chain Reaction and immunohistochemistry confirmed their differential expression, underscoring their significance in PCPG diagnosis and in predicting immunotherapy response. This study’s integration of amino acid metabolism-related genes into a risk prediction model offers critical clinical insights for PCPG risk stratification, potential immunotherapy responses, drug development, and treatment planning, marking a significant step forward in the management of this complex condition.

**Supplementary Information:**

The online version contains supplementary material available at 10.1007/s10528-024-10733-5.

## Introduction

Pheochromocytoma/Paraganglioma (PCPG) is a rare neuroendocrine tumor that originates from chromaffin cells in the adrenal medulla, with clinical manifestations that typically include hypertension, headache, palpitations, and profuse sweating (Lenders et al. 2005; Lam 2017). The incidence of PCPG is about 2–8 per million per year (Fassnacht et al. 2020). All PCPG are considered to have malignant potential, for which early surgical excision is the way to prevent metastasis (Dahia 2014). Despite advances in the genetics and transcriptomics of PCPG, therapeutic options for PCPG remain limited. In addition to surgery and radiation therapy, combination chemotherapy that includes cyclophosphamide-vincristine-dacarbazine (CVD) is recommended for advanced PCPG (Neumann et al. 2019; Pang et al. 2019). However, the CVD-based treatment is limited in improving the quality of life and the overall survival of PCPG patients (Huang et al. 2008). More importantly, since the pathogenesis of pheochromocytoma is complex, there is no effective targeted therapy to improve the progression of PCPG. Therefore, identification of new sensitive diagnostic biomarkers with PCPG or develop novel therapies for PCPG patients remains an urgent clinical need.

The metabolic ecology of tumors is complex, in which amino acid metabolism plays an important role in the energy production, nucleotide synthesis, cellular redox homeostasis, and connection between metabolic pathways (Martinez-Outschoorn et al. 2017; Vettore et al. 2020). In many tumors, the metabolic state of tumor cell is particularly affected by variations in amino acids and their derivatives (Sivanand and Vander Heiden 2020). For instance, leucine regulates the mTORC1 pathway by binding to Sestrin2, a negative regulator of rapamycin complex 1 (mTORC1), thereby affecting the metabolism and growth of tumors (Wolfson et al. 2016; Son et al. 2019; Sivanand and Vander Heiden 2020). Tumors depend on a large supply of amino acids to maintain sufficient energy during growth (Zhang et al. 2018; Pathria and Ronai 2021), such as synthesis/catabolism metabolism of glutamine, serine, and glycine has been considered as a metabolic regulator that supports tumor cell growth (Li and Zhang 2016). Altered tryptophan and arginine catabolism is one of the hallmarks of tumor immune microenvironment, as well as tumor growth can be slowed by increasing serine and glycine (Lemos et al. 2019; Muthusamy et al. 2020). Arginine derivatives are involved in processes related to RNA metabolism and regulate the function of specific T cells, which promote tumor growth and metastasis (Wu et al. 2021). However, the diagnosis and therapeutic value of amino acid metabolism-related biomarkers in PCPG has not been reported. Based on the interaction between amino acid metabolism and tumor immune microenvironment, it will be of great significance to explore the pathogenesis of amino acid metabolism and immune-related genes in PCPG patients and their potential application in immunotherapy.

In this study, we conducted bioinformatics analysis to explore the relationship between currently known amino acid metabolism-related genes and PCPG pathogenesis and immune infiltration. Our analysis aimed to identify potential diagnostic markers and therapeutic targets and to construct a risk model capable of predicting immunotherapy response in pheochromocytoma patients.

## Material and Methods

### Data Sources and Processing

The Amino acid metabolism-related genes were downloaded from the Molecular Signatures Database (http://www.gsea-msigdb.org/gsea/index.jsp) with the keyword “amino acid metabolism.” After removing the duplicated genes, 2357 amino acid metabolism-related genes remained (Supplementary Table 1).

The gene expression profiles of GSE19422 were downloaded from GEO database (https://www.ncbi.nlm.nih.gov/geo), including 6 normal samples and 84 tumor samples. We converted the probes into gene symbols for each dataset and in cases where multiple probes mapped to the same gene symbol, we selected the probe with the highest value as the gene expression value. The RNA-sequencing data of 3 normal samples and 184 PCPG samples were downloaded from TCGA database (https://portal.gdc.cancer.gov). TCGA database was applied to validate the model as an external validation dataset.

### Amino Acid Metabolism-Related Differentially Expressed Genes (DEGs) Screening

The R package “limma” was used to identify the DEGs with screening criteria set as |log2FoldChange|> 1 and adjusted *P*-value < 0.05. The Venn plot was used to visualize the intersection of DEGs and amino acid metabolism-related genes.

### Amino Acid Metabolism-Related DEGs Functional Enrichment Analysis

Gene Ontology (GO)/Kyoto Encyclopedia of Genes and Genomes (KEGG) and Disease Ontology (DO) enrichment analysis, as well as Gene Set Enrichment Analysis (GSEA), were performed using the “clusterProfiler” package of R software (version 4.1.3) to further investigate the potential biological functions of DEGs related to amino acid metabolism. The GO includes three ontologies: “Biological Process (BP),” “Cellular Component (CC),” and “Molecular Function (MF).” The KEGG was used to identify potentially important metabolic pathways. GSEA analysis enables enrichment analysis of individual metabolic pathways. The screening criteria were set at a false discovery rate (FDR) < 0.05.

### Construction of the Gene Co-expression Network

To explore the signature gene set of PCPG, weighted gene co-expression network analysis (WGCNA) was performed using R software (version 4.1.3) based on the R package “WGCNA.” WGCNA was used to explore the relationship between clinical features and expression modules. First, cluster the input samples and check whether the input samples and genes meet the conditions and build a co-expression network. Secondly, the optimal threshold is selected according to the R^2^ value and the slope value. Pearson correlation analysis was performed to construct an adjacency matrix and dissimilarity analyses were carried out. Next, the dynamic clipping tree analysis method was used to identify network modules and merge similar modules. Finally, correlation analysis was performed combining disease phenotype information.

### Gene Signature Construction

To analyze the most robust genes of PCPG-related molecules, the LASSO regression model was performed to further screen hub genes. Furthermore, the risk score is calculated by multiplying the gene expression by the regression coefficient obtained by Lasso regression. All patients were divided into high- and low-risk groups according to the median risk score. The ROC curve is generated by the “pROC” R package.

### Analysis of Immune Infiltration in PCPG

To evaluate the abundance of immune cell types between high-risk and low-risk samples in the dataset, the single-sample gene set enrichment analysis (ssGSEA), MCPCOUNTER, and ESTIMATE packages were used. The comparisons of the distributions between the two groups were made using the Wilcoxon signed-rank test. The immune cell composition in disease and normal samples was visualized using boxplots. All visualization work was done using R software. Differences were considered statistically significant when they showed a *P*-value < 0.05.

### Quantitative Real-Time Transcription (qRT)-PCR

A total of 18 pheochromocytoma tissue samples (stored in liquid nitrogen) were obtained with informed consent from 18 patients with pheochromocytoma undergoing surgery at the First Affiliated Hospital of Zhengzhou University. The research protocol of this study was approved by the Ethics Committee of the First Affiliated Hospital of Zhengzhou University (2022-KY-0209-002).

Total RNA was extracted from 9 pairs of clinical PCPG tumors and adjacent tissues (200 mg) using 1 mL of TRIzol reagent. Total RNA concentration was determined using NanoDrop2000. According to the manufacturer’s protocol, cDNA was obtained by reverse transcription using a reverse transcription Kit (PrimeScript™ RT reagent Kit with gDNA Eraser for qPCR). Quantitative real-time PCR amplification was performed with TB Green PCR master mixture (Takara, Japan) according to the manufacturer’s protocol.

Transcriptional expression was evaluated by the following primers: DDC, forward, 5′-TGGGGACCACAACATGCTG-3′, reverse, 5′-TCAGGGCAGATGAATGCACTG-3′ and SYT11, forward, 5′-GGGAAGGTGGACGTAGGAAC-30, reverse, 5′-GGGGTCAGGCTTGTAATAGGG-3′.

### Histopathology and Immunohistochemistry Staining

Paraffin-embedded tissue sections were prepared and sequentially defatted in xylene, rehydrated through a graded alcohol series, and stained with hematoxylin for 3 min using Leica ASP200S. Hematoxylin differentiation was applied for 45 s, followed by eosin staining for 2 min and sealing with a neutral gum (specified application method of Roche HE600).

For IHC, 3 μm sections were incubated at 65 °C for 2 h, defatted in xylene (2 × 15 min), and then rehydrated in graded alcohols (100% to 70%). Endogenous peroxidase was blocked with 3% hydrogen peroxide (10 min). After washing thrice with PBS, antigen retrieval was performed (specify conditions with thermal repair buffer). Sections were incubated in a chamber for 1 h, followed by overnight application of primary antibodies for DDC (1:1000, 10166-1-AP, Proteintech, Wuhan, China) and SYT11 (1:200, 12031-1-AP, Proteintech, Wuhan, China). A secondary antibody was applied for 1 h, staining initiated with DAB (specify duration), followed by sulforaphane application (Benchmark ultra, specify application method and duration). After sealing with neutral gum, staining patterns were observed microscopically.

### Bioinformatic Methods and Statistical Analysis

To assess the influence of amino acid metabolism on PCPG therapy, we evaluated the responsiveness of PCPG patients to anticancer drugs. Based on the Connectivity Map (cMAP, https://clue.io/) database, we utilized the “PharmacoG” R software package to estimate drug sensitivity in patients with PCPG. To evaluate the response of the disease to immunotherapy, the immunotherapy response was assessed using the Tumor Immune Dysfunction and Exclusion (TIDE, http://tide.dfci.harvard.edu/) database.

For the comparison of continuous variables between two groups, the independent Student’s *t* test was employed for normally distributed data, and the Mann–Whitney *U* test (Wilcoxon rank sum test) was used for non-normally distributed data. Correlation analyses were conducted using Spearman analysis. All data analyses were executed using R software (version 4.1.3) and relevant packages. *P* < 0.05 was considered statistically significant.

## Results

### Identification of Amino Acid Metabolism-Related Genes (AAMRGs) in Pheochromocytoma/Paraganglioma (PCPG)

**Fig. 1:**
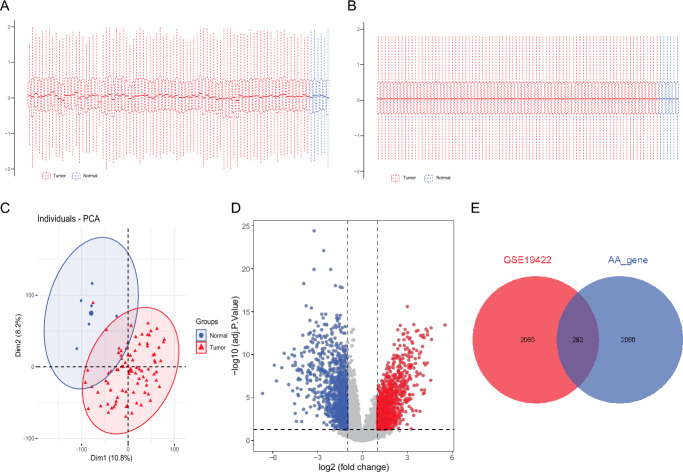
Identification of genes differentially associated with amino acid metabolism. **A** and **B** GSE19422 gene chip data before and after correction. **C** PCA analysis. **D** The volcano plot for differentially expressed genes (DEGs) between normal and PCPG samples in GSE19422. Red bubbles mean up-regulated genes, blue bubbles mean down-regulated genes, and gray bubbles mean non-significant genes. **E** The overlap of differentially expressed genes (DEGs) and amino acid metabolism-related genes was shown as a Venn diagram

In order to explore the potential molecular characteristics of the normal and disease groups, genes differentially expressed between the normal and disease groups were screened. We normalized the data to obtain biologically significant gene expression (Fig. 1A, B). PCA analysis was performed on the data which showed a biological significance between PCPG and normal samples (Fig. 1C). A total of 2357 differential genes were identified in the 6 normal and 84 PCPG samples from the GEO datasets (Fig. 1D). We intersected the differential genes with 2352 amino acid metabolism-related genes (AAMRGs) and 292 differential genes (AAMRGs) were obtained (Fig. 1E), including 131 up-regulated genes and 161 down-regulated genes (Supplementary Table 2).

### Functional Enrichment Analysis

**Fig. 2:**
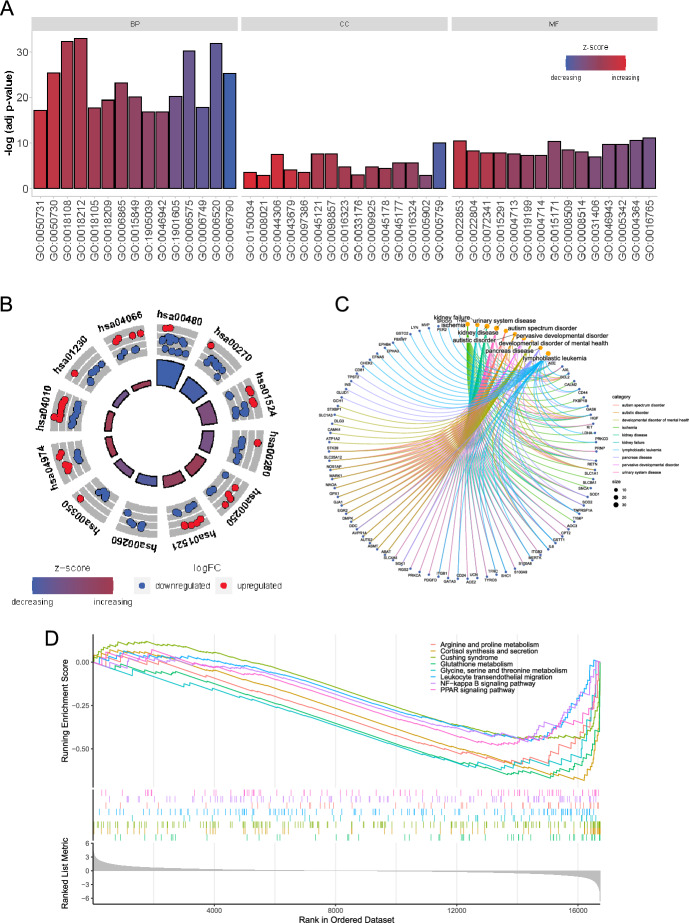
Expression patterns and biological significance of amino acid metabolism-related genes in PCPG. **A** First 15 items enriched for biological processes (BPs), cellular components (CCs), and molecular functions (MFs); the abscissa represents the GO term and ordinate is the -log (adj p-value). Band colors: blue represents downregulation and red represents upregulation. **B** KEGG pathways enriched by PCPG amino acid metabolism-related genes. **C** Disease Ontology (DO) pathways enriched by PCPG amino acid metabolism-related genes. **D** GSEA of clustering of eight items

For further exploration of the biological characteristics and significance of genes related to amino metabolism, we performed GO enrichment analysis, KEGG pathway enrichment analysis, DO enrichment analysis, and GSEA enrichment analysis. GO enrichment analysis showed significant correlations with tyrosine modification and phosphorylation, cellular amino acid metabolic processes, amino acid transport, and glutathione metabolic processes in biological process (BP) analysis. In molecular function (MF), it was significantly correlated with glutathione transferase activity, amino acid transmembrane transporter activity, organic acid transmembrane transporter activity, transmembrane receptor protein kinase activity, and protein tyrosine kinase activity. Cellular component (CC) enrichment analysis was found to be concentrated in the mitochondrial matrix, membrane microdomain, membrane rafts, and apical plasma membrane (Fig. 2A). KEGG pathway enrichment analysis mainly involved glutathione metabolism, cysteine and methionine metabolism, platinum drug resistance, degradation of valine, leucine and isoleucine, EGFR tyrosine kinase inhibitor resistance, tyrosine metabolism, and amino acid biosynthesis (Fig. 2B). Detailed results of the GO and KEGG enrichment analyses are shown in Supplementary Table 3 and Supplementary Table 4. DO enrichment analyses were mainly involved in renal failure, nephropathy, local ischemia, and urinary tract diseases (Fig. 2C).

Additionally, we performed GSEA analysis of all genes in the samples. We identified multiple metabolic processes, including arginine and proline metabolism, cortisol synthesis and secretion, Cushing’s syndrome, glutathione metabolism, leukocyte migration, NF-κB signaling pathway, and PPAR signaling pathway (Fig. 2D).

Taken together, these AAMRGs were enriched in the amino acid metabolic processes and transport and immunization pathway. Moreover, it is closely related to nephropathy and immune-related pathways. The above results could indicate that AAMRGs may affect the immune response and development of tumors by regulating amino acid metabolism.

### Weighted Gene Co-expression Network Analysis

**Fig. 3:**
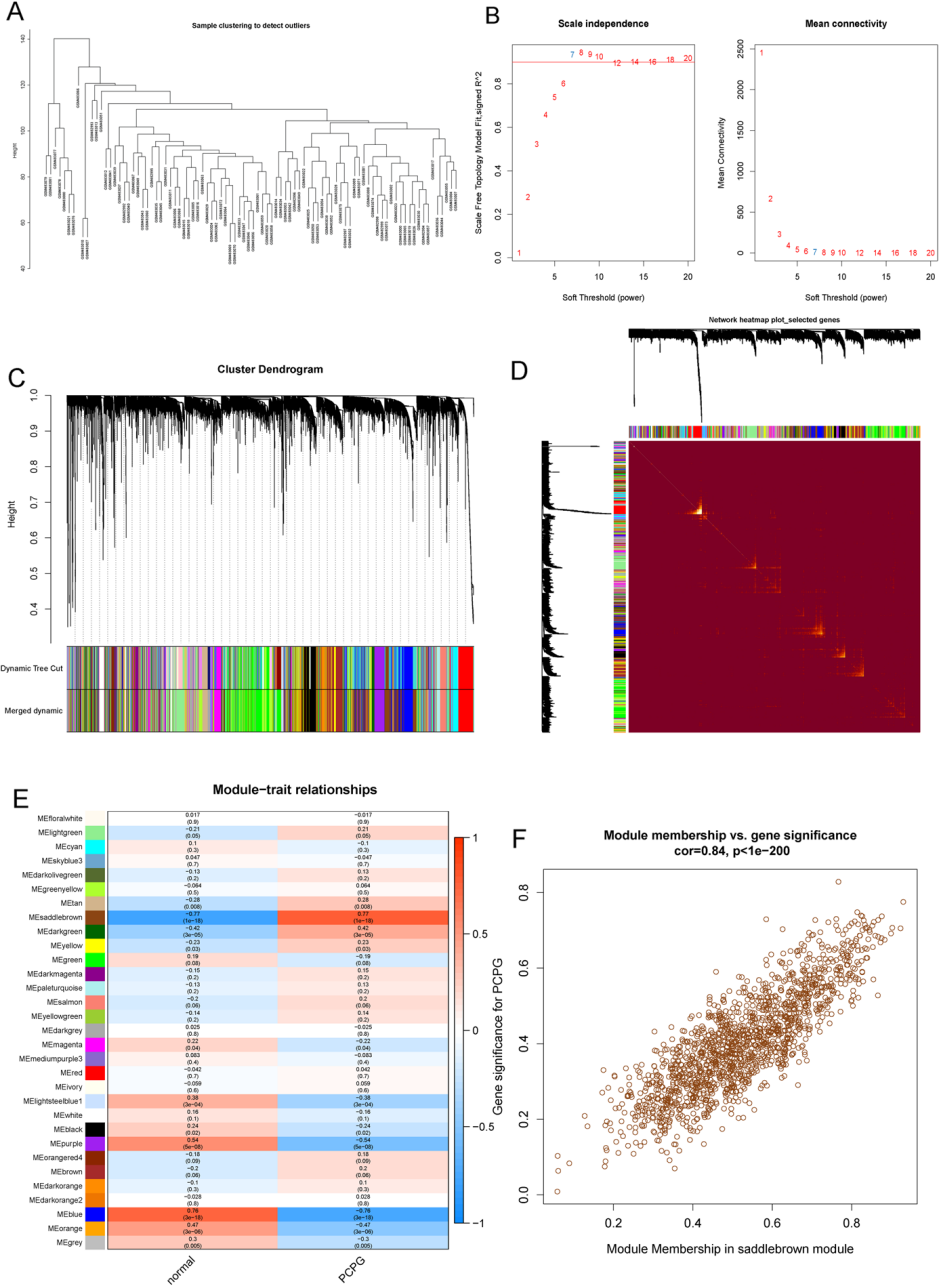
Weighted gene co-expression network analysis (WGCNA). **A** Sample dendrogram of genes between all samples. **B** Scale independence and average connectivity under distinct soft-thresholding powers. **C** Co-expression modules in each gene under the hierarchical clustering tree were assigned different colors. **D** TOM network clustering heatmap. **E** Heatmap of the relationship between co-expression modules and PCPG. **F** Correlation between module membership (MM) and genetic significance (GS) of the PCPG group

The weighted gene co-expression network (WGCNA) analysis was performed to explore pivotal genes that contribute to biological differences between patient populations. To screen genes, the absolute median difference (MAD) was used as a robust statistic that is more adaptable to outliers in the dataset than the standard deviation. Furthermore, hierarchical cluster analysis was performed on the samples to remove outlier samples. Network construction and module identification were then conducted using dynamic trimming tree analysis (Fig. 3A, C).

After determining the optimal soft threshold and building the scale-free network module with an R^2^ value of 0.93 and a slope of − 1.97 (Fig. 3B, D), we conducted a correlation analysis using scale-free network modules and external traits. From the resulting WGCNA traits heatmap, we selected the Module Eigengenes (ME) saddlebrown, which had the highest correlation with PCPG (|cor|= 0.77, *P*-value = 1e − 18) (Fig. 3E). The module membership and gene significance were highly correlated in the saddlebrown module (Fig. 3F). In summary, these results suggest a close correlation between PCPG and ME saddlebrown, which contains genes that may play an important function in the development of PCPG.

### Construction of Clinical Prediction Models

**Fig. 4:**
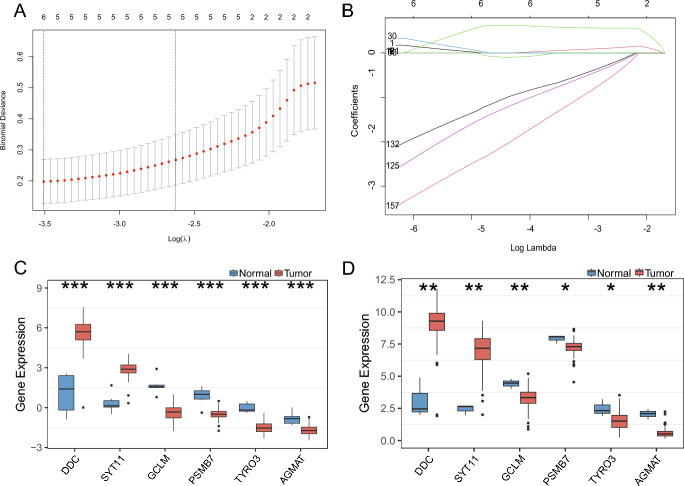
Development of amino acid metabolism-related gene signature for PCPG diagnosis. **A** Distribution of coefficients in the LASSO regression model. **B** Tuning feature selection in the LASSO model. **C** and **D** Differential expression levels of genes between tumor and normal in **C** GEO and **D** TCGA cohorts

To further identify the best candidate genes, logistic least absolute shrinkage and selection operator (LASSO) regression analysis algorithm was used to filter the six optimal variables from the variables of AAMRGs in the saddlebrown module described above using ten-fold cross-validation. An optimal prediction model containing six non-zero coefficient genes (DDC, SYT11, GCLM, PSMB7, TYRO3, and AGMAT) was constructed when the model reached the minimum value of lambda (λ) (Fig. 4A, B). The Risk Score is as follows: risk score = (0.051*the expression of DDC)+(0.586*the expression of SYT11) − (0.758*the expression of GCLM) − (0.658*the expression of PSMB7) − (1.313*the expression of TYRO3) − (0.001*the expression of AGMAT). Compared with the normal group, the expression of DDC and SYT11 were remarkably up-regulated in the tumor group, while the expression of GCLM, PSMB7, TYRO3, and AGMAT were remarkably down-regulated in the tumor group (Fig. 4C). Moreover, the expression levels of these six genes were verified in the TCGA dataset (Fig. 4D). Spearman algorithm was used to analyze the correlation and interaction between amino acid-related genes (Additional file 5. Fig. S1). Patients were divided into high-risk and low-risk groups based on median risk scores. In short, the six amino acid metabolism-related features screened may play a key role in the pathogenesis of PCPG disease.

### Identification of Immune Microenvironment and Biological Function Characteristics of Different Risk Subtypes

**Fig. 5:**
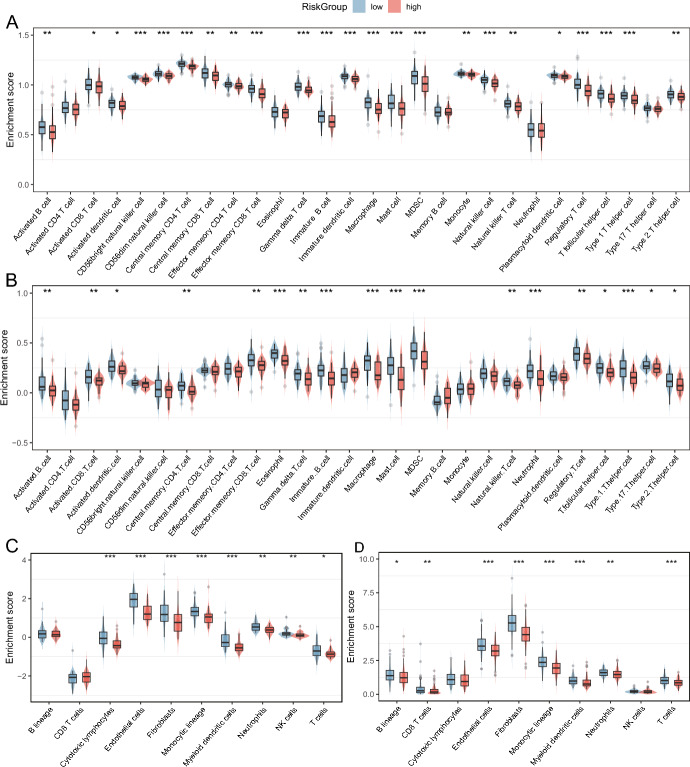
Analysis of immune infiltration in PCPG. **A** and **B** Enrichment fractions of immune cells in the high- and low-risk groups of PCPG patients were assessed by ssGSEA and MCPCOUNTER. *P* values were calculated using Wilcoxon test for two groups. Differential enrichment scores of 28 immune cell signatures between the high- and low-risk groups in **A** GEO and **B** TCGA cohorts. **C** and **D** Differential absolute abundance of 8 immune cells and 2 stromal cells between the high- and low-risk groups in the **C** GEO and **D** TCGA cohorts (*P < 0.05, **P < 0.01, ***P < 0.001)

We conducted a comprehensive analysis of immune infiltration differences between high-risk and low-risk samples using three distinct approaches: single-sample gene set enrichment analysis (ssGSEA), MCPCOUNTER, and ESTIMATE. Specifically, we utilized the ssGSEA algorithm to evaluate the abundance of 28 immune cell species between high-risk and low-risk groups. The resulting immune cell ratios for 28 PCPG are illustrated in Fig. 5A. Compared with the high-risk group, we observed significantly higher infiltration levels of various immune cell types, including Activated B cells, Activated CD8 T cells, Central memory CD4 T cells, Effector memory CD8 T cells, Gamma delta T cells, Eosinophils, Mature B cells, macrophages, mast cells, NK cells, neutrophils, regulatory T cells, and TFH cells, in the low-risk group (Fig. 5A). MCPCOUNTER is a marker-based genetic method for quantifying tumor-infiltrating immune cells. The abundance score for each cell type was calculated independently for each sample, based on the geometric mean of the cell type-specific gene expression values. MCPCOUNTER analysis results showed that T cells, cytotoxic lymphocytes, NK cells, Monocytic lineage, Myeloid dendritic cells, Neutrophils, Endothelial cells, and Fibroblasts had higher enrichment fractions in the low-risk group (Fig. 5C). ESTIMATER results indicate that AAMRGs risk scores tend to be negatively correlated with the level of immune cell infiltration (Additional file 6. Fig. S2A–D). Similarly, in the TCGA cohort, the ssGSEA results showed that the low-risk group had higher levels of Activated B cell, Activated CD8 T cell, Central memory CD4 T cell, Effector memory CD8 T cell, Gamma delta T cell, Immature B cell, and Macrophage, and Mast cell infiltration levels were higher (Fig. 5B). Compared to the GEO cohort, the results of MCPCOUNTER analysis in the TCGA cohort showed higher enrichment fractions of T cells, CD8 T cells, Monocytic lineage, Myeloid dendritic cells, Neutrophils, Endothelial cells, and Fibroblasts in the high-risk group (Fig. 5D). Overall, the results indicate that amino acid metabolism-related signatures were closely associated with the tumor immune microenvironment that may lead to differences in immune infiltration between the two risk subgroups.

**Fig. 6:**
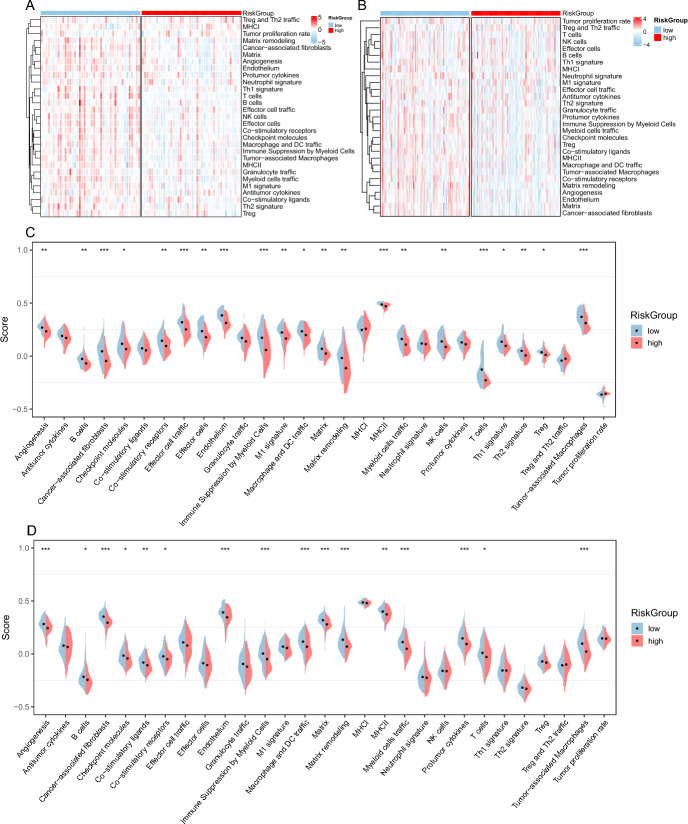
Gene set variation analysis (GSVA) in PCPG. **A** and **B** Heatmaps showed the overall characteristics of 29 immune features between the GEO (**A**) and TCGA (**B**) cohorts of PCPG risk subtypes. **C** and **D** Differential expression of 29 signatures between the PCPG risk subtypes in GEO (**C**) and TCGA (**D**) cohorts. *P*-values were calculated using Wilcoxon test for two groups (*P < 0.01, **P < 0.001, ***P < 0.001)

We investigated the immune-related mechanisms underlying high- and low-risk groups in PCPG patients by conducting a gene set variation analysis (GSVA) enrichment analysis based on the gene sets provided by Bagaev et al. (2021). The results indicated that the low-risk group had significantly higher enrichment of immune-related pathways, including B cells, Co-stimulatory receptors, Effector cell traffic, MHCII, and T cells, compared to the high-risk group (Fig. 6A, C). In contrast, the high-risk group exhibited reduced activation of these pathways (Fig. 6B, D), indicating a weakened immune response against tumor cells. Therefore, targeting amino acid metabolism-related genes may represent a potential strategy to modulate the immune microenvironment and enhance the efficacy of immunotherapy in PCPG patients.

### Association Between High- and Low-Risk Groups of PCPG and Immune Modulators

**Fig. 7:**
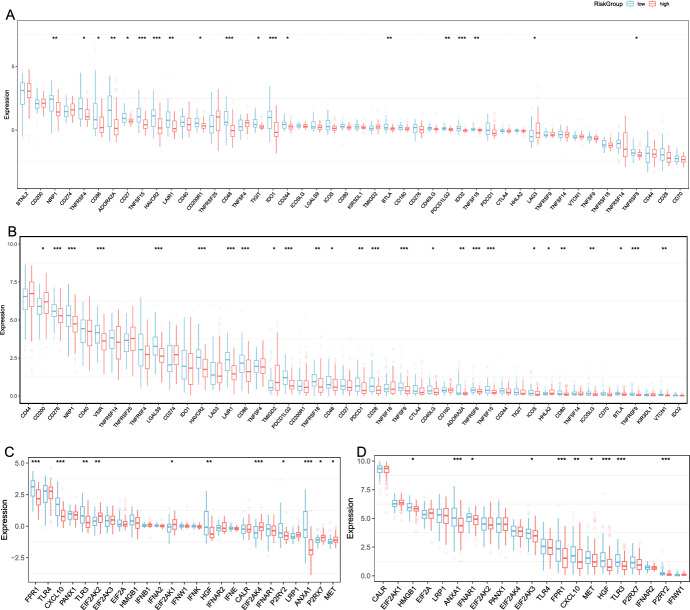
Association between high- and low-risk groups and ICPs and ICD modulators. **A** and **B** Differential expression of ICP genes between the high- and low-risk groups in **A** GEO and **B** TCGA cohorts. **C** and **D** Differential expression of ICD modulator genes between the high- and low-risk groups in **C** GEO and **D** TCGA cohorts. *P*-values were calculated using Wilcoxon test for two groups (*P < 0.01, **P < 0.001, ***P < 0.001)

Given that immune checkpoints (ICPs) and immunogenic cell death (ICD) regulators function as important immune regulators in tumors, we compared the expression levels of immune checkpoints in the high-risk and low-risk groups. A total of 46 ICP-related genes were analyzed in GEO and TCGA cohorts, of which 19 (41.3%) genes in the GEO cohort (Fig. 7A) and 26 (56.5%) genes in the TCGA cohort (Fig. 7B) had differential expression between the high-risk groups and low-risk groups. For instance, NRP1, TNFRSF4, CD86, ADORA2A, CD27, TNFSF15, HAVCR2, LAIR1, CD200R1, CD48, TIGIT, IDO1, CD244, BTLA, PDCD1LG2, IDO2, TNFSF18, and TNFRSF8 were significantly up-regulated in the low-risk group, while CD276, NRP1, VSIR, LGALS9, HAVCR2, LAIR1, CD86, PDCD1LG2, TNFRSF18, CD48, PDCD1, CD28, TNFSF9, CD40LG, ADORA2A, TNFRSF8, TNFSF15, ICOS, HHLA2, CD80, ICOSLG, BTLA, TNFRSF9, and VTCN1 were overexpressed in the low-risk group in the TCGA cohort. The results of our analysis imply that the amino acid metabolism-related signature may have potential clinical utility in evaluating PCPG immunotherapy. 25 ICD genes were detected in the GEO cohort, of which 11 (44.0%) were differentially expressed between the two groups (Fig. 7C). For example, the expression of FPR1, CXCL10, TLR3, EIF2AK2, EIF2AK1, HGF, EIF2AK4, P2RY2, ANXA1, P2RX7, and MET were all higher in the low-risk group than in the high-risk group. While there were 21 ICD genes expressed in the TCGA cohort, 10 (47.6%) were significantly different between the high-risk groups and low-risk groups (Fig. 7D). For example, HMGB1, ANXA1, IFNAR1, EIF2AK3, FPR1, CXCL10, MET, HGF, TLR3, and P2RY2 were overexpressed in the low-risk group than in the high-risk group. The expression of ICD regulators was significantly higher in the low-risk group than in the high-risk group, which indicated a relatively higher level of immunoreactivity in the low-risk group. Therefore, the risk model could reflect the expression levels of ICPs and ICD regulators, which are immune-related genes that may be involved in the immune regulation of PCPG.

In brief, the low-risk group showed elevated expression of ICP and ICD modulators, but exhibited lower sensitivity to immune checkpoint inhibitors. Moreover, the low-risk group had a greater ability to evade the immune system. These results again validated that amino acid metabolism-related signatures play an important regulatory role in forming different immune microenvironments in PCPG patients.

### Immunotherapy and Potential Drugs with Potential Activity for PCPG

**Fig. 8:**
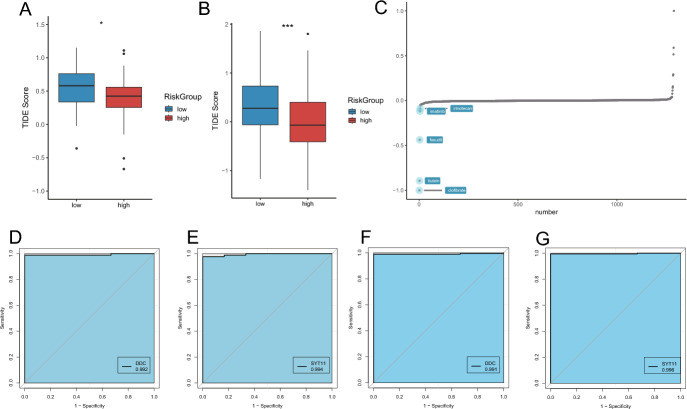
Risk model predicts treatment outcomes and potentially active drugs in PCPG patients. **A** and **B** Prediction of immunotherapy in high- and low-risk groups of PCPG patients in the **A** GEO and **B** TCGA cohorts. **C** Potential drug prediction on data from GEO19422. **D** and **E** ROC curves of DDC (**D**) and SYT11 (**E**) genes in GEO cohort. **F** and **G** ROC curves of DDC (**F**) and SYT11 (**G**) genes in TCGA cohort

To identify potential populations that could benefit from immunotherapy, the TIDE algorithm was employed to predict the response to immunotherapy for different risk subtypes. The results indicated that, in the GEO cohort, the high-risk group exhibited a significantly better response to immunotherapy than the low-risk group (Fig. 8A). The same result was also observed in TCGA, where the high-risk group benefited more from immunotherapy (Fig. 8B). The “PharmacoG” R package was used to identify drugs with potential activity in PCPG to identify potential drugs. The prediction of drugs was performed with 292 DEGs related to amino acid metabolism as input genes. As the results showed the top five drugs with enrichment scores were clofibrate, butein, fasudil, imatinib and irinotecan (Fig. 8C). The results suggest that these drugs may have better clinical efficacy in PCPG patients.

**Fig. 9:**
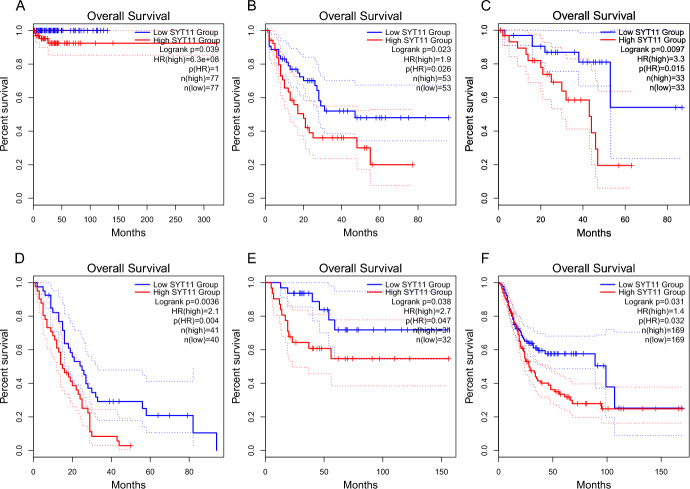
Relationship between expression characteristics of SYT11 and prognosis. **A** and **C** Kaplan–Meier curves showing overall survival (OS) of patients with PCPG (**A**), Acute Myeloid Leukemia (**B**), and Melanoma (**C**) stratified based on SYT11 expression levels. **D** and **F** Kaplan–Meier curves showing overall survival (OS) of patients with Mesothelioma (**D**), Adrenocortical Cancer (**E**) and Bladder Cancer (**F**) stratified based on SYT11 expression levels

Besides, our predictive model performed effectively in assessing the diagnosis of patients with pheochromocytoma. The diagnostic performance of DDC and SYT11 was assessed by calculating the area under ROC curve (AUC) in the validation sets of TCGA and GSE39716. In the TCGA dataset, the AUC value of DDC was 0.991 (Fig. 8F), while that of SYT11 was 0.996 (Fig. 8G), indicating excellent diagnostic accuracy. In the GSE39716 dataset, the AUC values of DDC and SYT11 were 0.84 and 0.67, respectively, indicating moderate diagnostic performance (Additional file 7. Fig. S3A, B). In addition, survival analysis revealed that high expression of SYT11 in PCPG patients was associated with a poor prognosis (Fig. 9A). Notably, high SYT11 expression in other cancer types, such as Adrenocortical Cancer, Bladder Cancer, Acute Myeloid Leukemia, Mesothelioma, and Ocular Melanomas, also associated with poor prognoses (Fig. 9B–F). These findings indicate that DDC and SYT11 have promising potential as diagnostic biomarkers in PCPG.

In summary, the analysis of amino acid metabolism-related genes has revealed their potential as valuable biomarkers for the diagnosis of PCPG. Furthermore, our study suggests that amino acid metabolic risk grouping can effectively stratify patients and predict treatment outcomes, particularly in the context of immunotherapy and drug screening.

### Validation of DDC and SYT11 as Key Biomarkers in PCPG

**Fig. 10:**
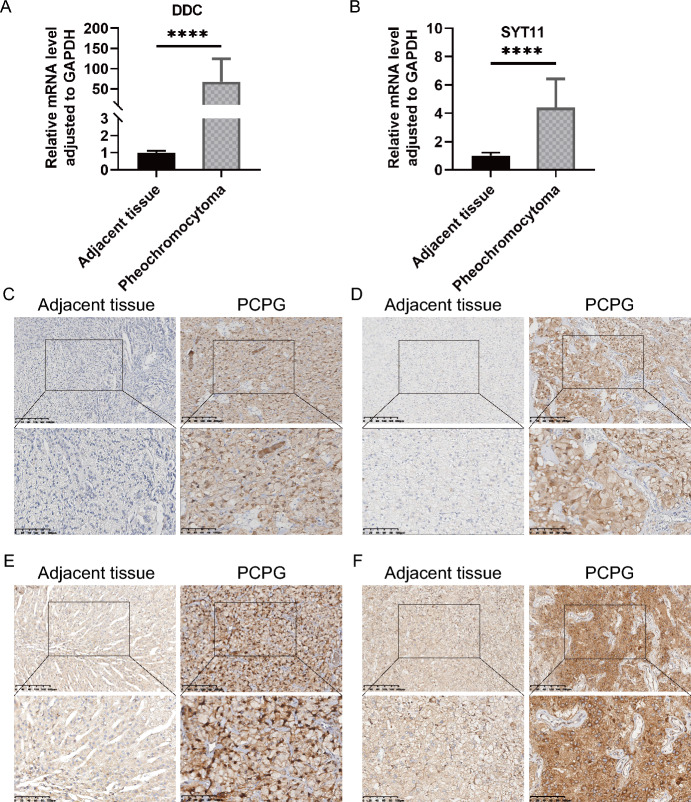
Validation of DDC and SYT11 expression at the mRNA and protein levels in PCPG. **A** Relative expression levels of DDC in PCPG and adjacent normal tissues. **B** Relative expression levels of SYT11 in PCPG and adjacent normal tissues. **C** and **D** Representative IHC images of DDC in PCPG tissues and adjacent tissues. **E** and **F** Representative IHC images of SYT11 in PCPG tissues and adjacent tissues

We evaluated the expression levels of AAMRGs, specifically DDC and SYT11, in PCPG and adjacent tissues. Our findings indicated a substantial upregulation of DDC and SYT11 in PCPG tissues compared with adjacent tissues (Fig. 10A, B). Furthermore, immunohistochemistry staining analyses substantiated a significant enhancement in the expression of DDC and SYT11 in PCPG tissues relative to adjacent and normal adrenal tissues (Fig. 10C–F and Additional File 8. Fig. S4A–H). Collectively, these results corroborate our previous analyses concerning the expression of DDC and SYT11.

## Discussion

Tumor metabolic reprogramming endows cancer cells proliferation and viability. At present, dysregulated metabolism/catabolism of amino acids, particularly glutamine, glycine and serine, have been identified to support the metabolic regulators of cancer cell growth (Li and Zhang 2016). In addition, amino acids are involved in the remodeling of tumor microenvironment and the maintenance of tumor redox balance. For instance, Kynurenine produced from tryptophan induces immune suppression of tumors by binding and activating transcription factors (Lieu et al. 2020). However, the impact of genes related to amino acid metabolism in the immune microenvironment and immunotherapy of pheochromocytoma have not been clarified.

In this study, a gene set consisting of 2357 genes involved in amino acid metabolism was defined, utilizing the Molecular Signatures database. We screened 292 differentially (DEGs) related genes of amino acid metabolism. Subsequently, GO annotation and KEGG enrichment analyses indicated that these DEGs were mainly involved in the amino acid metabolic processes and transport and immunization pathway. Then, we constructed amino acid metabolism profiles by WGCNA and LASSO regression model for investigating the impact of amino acid metabolism on pheochromocytoma pathogenesis and immunity and identified six hub genes (DDC, SYT11, GCLM, PSMB7, TYRO3, and AGMAT). The TCGA dataset confirmed that DDC and SYT11 were highly expressed in PCPG and were highly similar in biological function (Fig. 5D), while GCLM, PSMB7, TYRO3, and AGMAT were down-regulated in PCPG. We revealed two distinct risk subgroups based on the expression of 6 amino acid metabolism-related genes. Notably, significant differences in immune activities were observed between PCPG patients with different amino acid metabolism patterns. Furthermore, according to different immune infiltration algorithms, we found that the infiltration patterns of immune cells varied in different subgroups, and immune cells showed a significant negative relationship with the risk score of PCPG patients. Compared with high-risk patients, low-risk patients presented with higher levels of immune infiltration. As a consequence, high-risk patients might benefit from immunotherapy. The above results suggest that the amino acid metabolism-related signature have excellent and reliable predictive power for the diagnosis and immunotherapy of PCPG patients.

Several studies have reported a link between six central genes that are, to some extent, involved in the process of PCPG. DDC, SYT11, GCLM, PSMB7, TYRO3, and AGMAT are the key genes used to construct the amino acid metabolism-related signature. DDC was considered a biomarker in different tumors, such as neuroendocrine tumors, neuroblastoma, and prostate cancer (Kontos et al. 2010; Koutalellis et al. 2012). High expression of DDC was also observed in breast cancer, highly differentiated colorectal tumors, small cell lung cancer, neuroblastoma, and pheochromocytoma (Kontos et al. 2010; Tremmel et al. 2020). SYT11 is a member of the family of synaptic lectins (SYTs), which are known calcium sensors and mediate calcium-dependent regulation of membrane transport in synaptic transmission. A recent study revealed that SYT11 driven the invasion and metastasis of lung cancer by altering the tumor microenvironment (Bajaj et al. 2022). SYT11 is highly expressed in diffuse gastric cancer, which plays a crucial role in the JNK11 phosphorylation and cell proliferation and metastasis (Kim et al. 2022). Furthermore, the elevated expression of the SYT11 gene has been associated with a poorer prognosis in various tumor types, providing additional evidence to support its potential as a diagnostic marker for PCPG. As a summary, the amino acid metabolism-related pivotal genes DDC and SYT11 could potentially be a novel biomarker to reveal the pathogenesis of PCPG.

GCLM is the key velocity-limiting enzyme for glutathione synthesis, which was essential for maintaining the intracellular redox state (Schaupp et al. 2022). When glutathione deficiency leads to increased oxidative stress, causing abnormal redox signaling to initiate and promote tumor progression (Luo et al. 2022). Proteasome subunit beta-7 (PSMB7) is a member of the 20S and 26S proteasome complexes, which play a key role in maintaining protein homeostasis (Rho et al. 2008; Houhou et al. 2019). In addition to exhibiting significantly lower expression levels in tumor cells, PSMB7 actively participates in the generation of MHC Class I-presenting antigen peptides by forming the 20S-PA28 complex (Groettrup et al. 1996; Eang et al. 2009). TYRO3 promoted cell growth and metastasis through Wnt/β-catenin signaling-mediated EMT in gastric cancer, in which TYRO3 silencing distinctively suppressed gastric cancer cells proliferation, invasion, and metastasis. (Chen et al. 2020; Uejima et al. 2020). Moreover, TYRO3 increased CD8+T cell infiltration by modulating MDSC function, which enhanced the effect of anti-PD-1 checkpoint inhibitor immunotherapy (Holtzhausen et al. 2019). AGMAT was produced from the decarboxylation of L-arginine as a primary amine, which has been documented to have widely biological effects, including inhibition of cell proliferation and stimulation of glomerular filtration rate (Benítez et al. 2018). The findings indicate that GCLM, PSMB7, TYRO3, and AGMAT potentially serve as protective genes in PCPG. Moreover, they may play significant roles in shaping the tumor microenvironment and influencing the effectiveness of immunotherapy in PCPG.

The tumor microenvironment (TME) is mainly composed of tumor cells, immune cells, and extracellular matrix, which is closely related to cancer development, growth, and metastasis (Hanahan and Coussens 2012; Liu et al. 2023). Studies have shown that the infiltration density and function of immune cells in TME profoundly affect tumor progression and immunotherapy (Quail and Joyce 2013; Gasser et al. 2017). Based on the results of the enrichment analysis, we can reasonably infer that amino acid metabolism-related genes were related to the tumor immune microenvironment. Therefore, we explored the immune infiltration in high- and low-risk groups by the ssGSEA method. We found that high-risk patients had lower levels of antitumor-infiltrating immune cells, whereas, the low-risk group had higher populations of antitumor immune cells in the tumor microenvironment (e.g., B cells, CD8 T cells, NK cells, dendritic cells (DCs), mast cells, neutrophils, T follicular helper cells), which indicated globally impaired immune function in the GEO cohort of high-risk patients. Similar conclusions were validated in the TCGA cohort. Interestingly, MDSC and Treg cell expression were elevated in low-risk patients with high antitumor activity, which may be attributed to TME containing a large number of immunosuppressive factors that greatly inhibit the antitumor function of immune cells. Furthermore, the increase of Treg cells may inhibit the antitumor immune response and affect the effectiveness of immunotherapy (Tanaka and Sakaguchi 2017; Kaminskiy et al. 2022). Investigation has shown that amino acid metabolism can affect specific immune responses by modulating tumor immune infiltrating cells (Nakamura et al. 2007; Timosenko et al. 2017; Lemos et al. 2019). According to the tumor immune editing hypothesis, there were both antitumor immune responses and promotion of tumor escape or immune destruction in cancer (Dunn et al. 2002). Moreover, immune scores derived from the MCPCOUNTER algorithm confirmed low immunogenicity in the high-risk group. AAMRGs risk scores tended to be negatively correlated with the level of immune cell infiltration (Fig. S2A-D), which indicates the increased AAMRGs risk was associated with decreased stromal cells and immune cells (Yoshihara et al. 2013). As a result, our risk score could inform the immune infiltration of the PCPG tumor microenvironment and might influence immunotherapy.

Given that ICP and ICD modulators are factors in the effect of immunotherapy, we further analyzed the expression of ICP and ICD in different risk subgroups. In the GEO and TCGA cohorts, ICP and ICD modulators differed significantly in the risk groups, implying that risk scores may inform immunotherapy in patients with different immune status. On the other hand, increased expression of ICD modulators in tumors also suggests that mRNA vaccines have greater potential in these tumors. In addition, we also used the TIDE database for immunotherapy prediction. The TIDE score can be used as an alternative biomarker to predict the response to immune checkpoint blockade by calculating the TIDE score for each tumor sample (Fu et al. 2020). The TIDE results confirmed better immunotherapy efficacy and greater sensitivity in the high-risk group compared to the low-risk group. The efficacy of immunotherapy is often hindered by various immunosuppressive signals present within the tumor microenvironment (TME) (Smyth et al. 2016). The analysis of Gene set variation analysis (GSVA) demonstrated that the low-risk group displayed elevated activity in crucial factors, including Tumor-associated macrophages, Angiogenesis, and Matrix remodeling. Research indicates that Tumor-associated Macrophages have the ability to establish and reshape the extracellular matrix structure, enabling tumor cells to invade through the tumor microenvironment (TME) and thereby affecting the effectiveness of immunotherapy (Noy and Pollard 2014; Mantovani et al. 2017).

To identify drugs that may benefit PCPG, we performed drug prediction using “PharmacoG” R package based on amino acid metabolism-related gene expression. The analysis identified clofibrate, butein, fasudil, imatinib, and irinotecan as the most significant potential therapeutic agents for treating pheochromocytoma. Clofibrate attenuated the inflammatory response by activating the PPARα pathway to inhibit the growth of human breast cancer cells (Chandran et al. 2016). Butein causes cell cycle arrest, apoptosis, and invasion in non-small cell lung cancer cells via activation of endoplasmic reticulum stress and ROS pathway (Di et al. 2019). The application of butein led to the downregulation of hTERT gene expression, effectively suppressing the proliferation and differentiation of human leukemia cells (Moon et al. 2009). After the fasudil treatment, breast cancer cells were significantly reduced in terms of migration and partial disorganization of actin filaments, which resulted in reduced invasive abilities in vitro (Guerra et al. 2017). Imatinib provides better therapeutic effect for leukemia, which has the effect of inhibiting tumor cell proliferation and promoting tumor cell apoptosis in leukemia (Lompardía et al. 2019). Irinotecan has been shown to have a significant effect in colorectal cancer, acting specifically in the S phase of the cell cycle, by blocking DNA synthesis and inhibiting the growth of tumor cells (Matsusaka and Lenz 2015; Fornaro et al. 2019).

Overall, we have developed a new risk model of PCPG and using the AAMRG risk model that can help diagnose patients with pheochromocytoma and could help reveal the interactions between amino acid metabolism and the tumor microenvironment. The model will help to select high-risk patients and provide new directions for immunotherapy prediction and potential drug screening.

## Conclusion

In conclusion, our study established a predictive signature associated with amino acid metabolism that can accurately distinguish PCPG patients with different clinical outcomes. Moreover, this study provides evidence to support the combination of amino acid metabolism and immunotherapy for future clinical treatment of PCPG patients.

## Supplementary Information

Below is the link to the electronic supplementary material.

Supplementary file1 (CSV 133 kb)—Table 1. Amino acid metabolism-related genes.

Supplementary file2 (CSV 34 kb)—Table 2. 292 differential amino acid metabolism-related genes.

Supplementary file3 (CSV 314 kb)—Table 3. The complete results of GO analysis.

Supplementary file4 (CSV 43 kb)—Table 4. The complete results of KEGG analysis.

Supplementary file5 (PDF 9 kb)—Figure S1 Correlation heatmap depicting the relationships between six hub genes. The association between amino acid-related genes.

Supplementary file6 (PDF 682 kb)—Figure S2 Estimate analysis for PCPG patients. (A) The relationship between risk score and stromal score in the GEO cohort. (B) The relationship between risk score and immune score in GEO cohort. (C) The relationship between risk score and stromal score in the TCGA cohort. (D) The relationship between risk score and immune score in the TCGA cohort

Supplementary file7 (PDF 94 kb)—Figure S3. ROC curves of DDC and SYT11 in the GSE39716 cohort.

Supplementary file8 (PDF 7772 kb)—Figure S4 Additional Validation of DDC and SYT11 Protein Expression Through Immunohistochemistry. (A-D) Additional representative images of IHC staining showing DDC protein expression in PCPG. (E-H) Additional representative images of IHC staining showing SYT11 protein expression in PCPG.

## Data Availability

The datasets generated during and/or analyzed during the current study are available from the corresponding author on reasonable request. All databases are accessible via the following respective websites: For TCGA, one can visit (https://portal.gdc.cancer.gov/) and for GEO, the link is (https://www.ncbi.nlm.nih.gov/geo/).

## Abbreviations

PCPG: Pheochromocytoma/paraganglioma
AAMRGs: Amino acid metabolism-related genes
GEO: Gene expression omnibus
WGCNA: Weighted gene co-expression network analysis
TCGA: The cancer genome atlas
LASSO: Least absolute shrinkage and selection operator
ROC: Receiver operating characteristic
AUC: Area under the curve
KEGG: Kyoto encyclopedia of genes and genomes
GO: Gene ontology
BP: Biological processes
CC: Cellular components
MF: Molecular functions
GSVA: Gene set variation analysis
GSEA: Gene set enrichment analysis
ssGSEA: Single-sample GSEA
TME: Tumor microenvironment
DO: Disease ontology
TIDE: Tumor immune dysfunction and exclusion
cMAP: Connectivity map
ICP: Immune checkpoints
ICD: Immunogenic cell death
