# Supplementary figures and images for: Constructing a Novel Amino Acid Metabolism Signature: A New Perspective on Pheochromocytoma Diagnosis, Immune Landscape, and Immunotherapy
Source: Biochem Genet. 2024 Mar 25;63(1):850–74. doi: 10.1007/s10528-024-10733-5 (PMC11832799; doi:10.1007/s10528-024-10733-5)

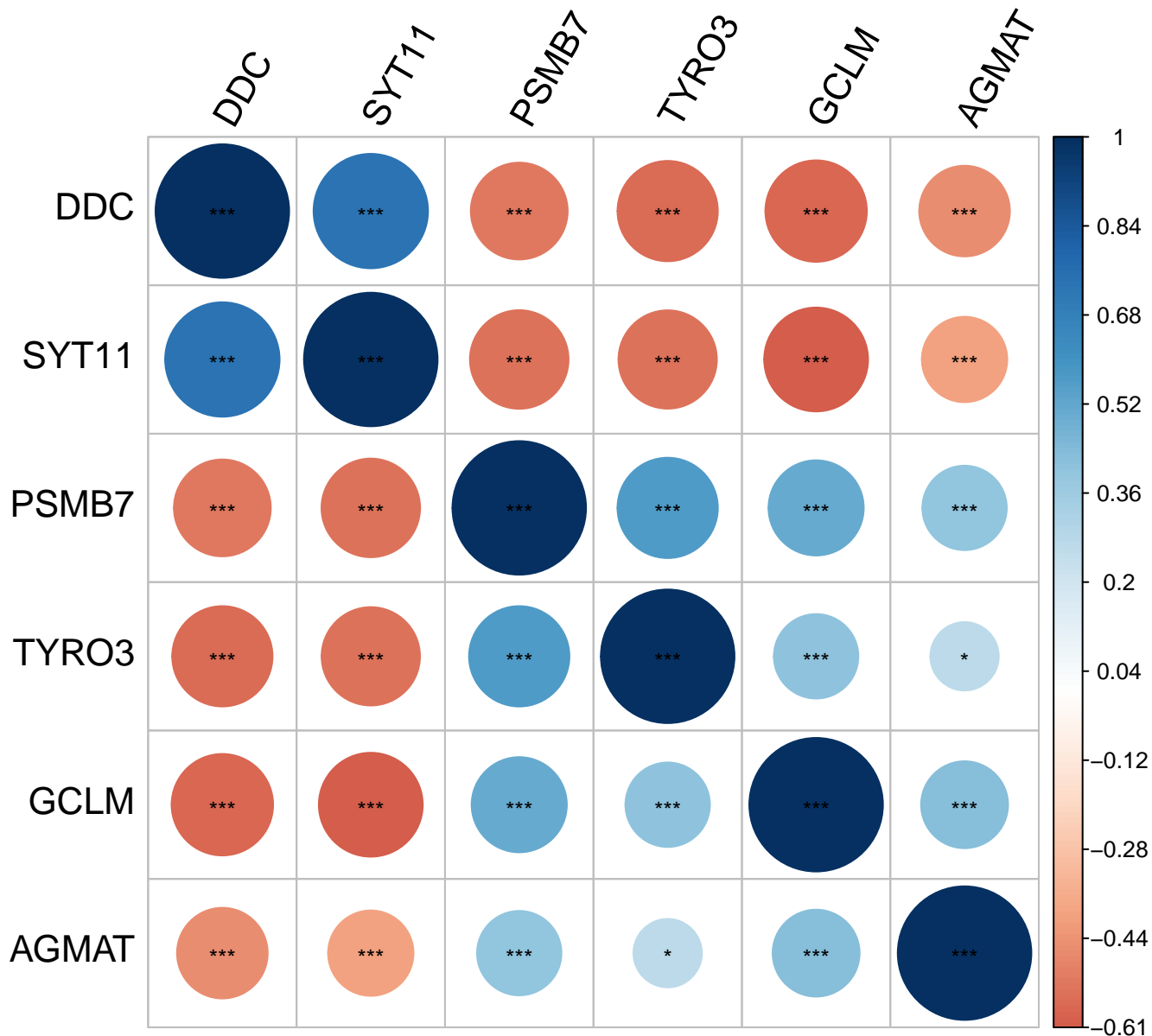

Supplement: Supplementary file 5 — Supplementary file5 (PDF 9 kb)—Figure S1 Correlation heatmap depicting the relationships between six hub genes. The association between amino acid-related genes. [file 10528_2024_10733_MOESM5_ESM.pdf]

A

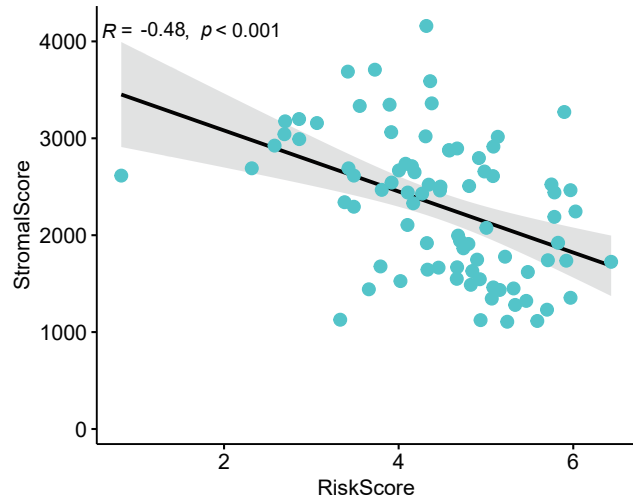

B

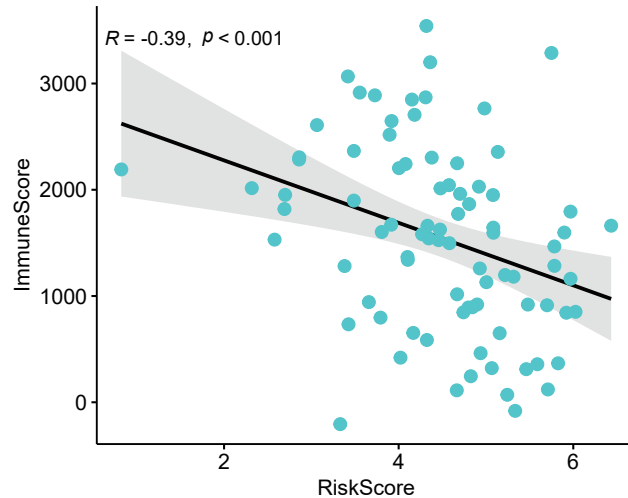

C

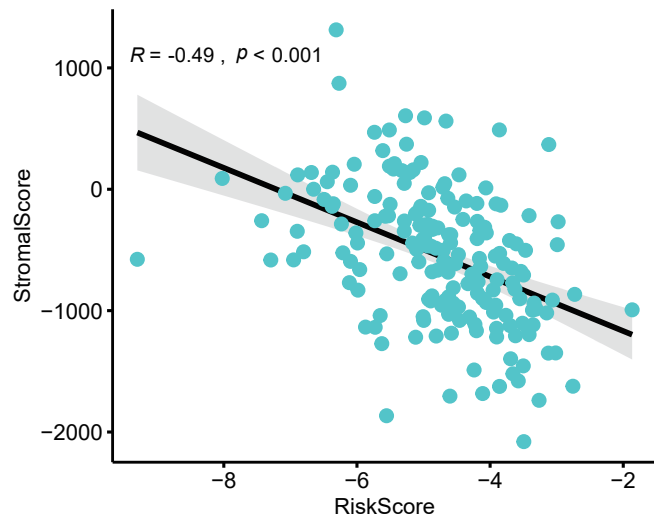

D

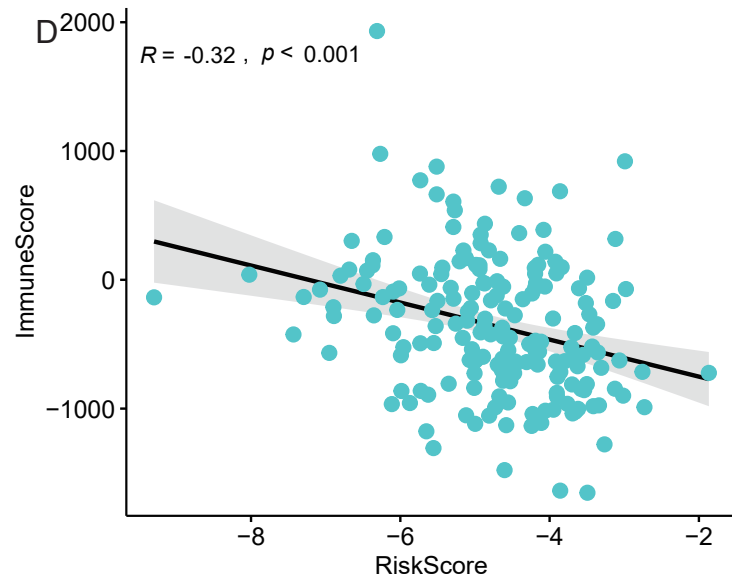

Supplement: Supplementary file 6 — Supplementary file6 (PDF 682 kb)—Figure S2 Estimate analysis for PCPG patients. (A) The relationship between risk score and stromal score in the GEO cohort. (B) The relationship between risk score and immune score in GEO cohort. (C) The relationship between risk score and stromal score in the TCGA cohort. (D) The relationship between risk score and immune score in the TCGA cohort [file 10528_2024_10733_MOESM6_ESM.pdf]

**A**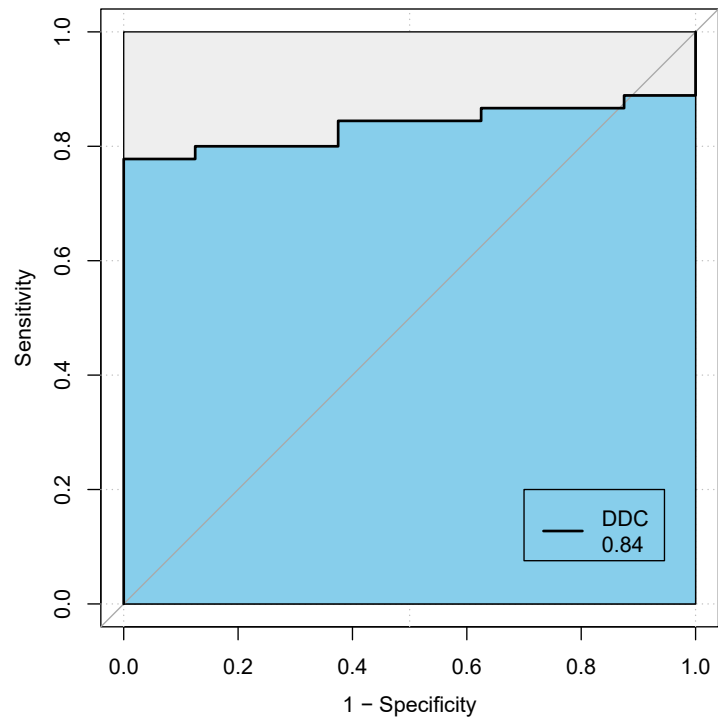**B**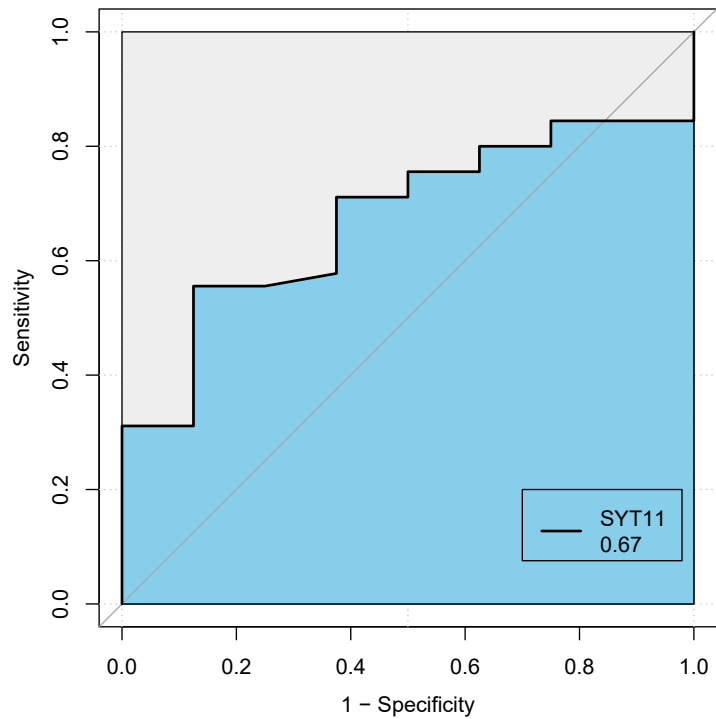

Supplement: Supplementary file 7 — Supplementary file7 (PDF 94 kb)—Figure S3. ROC curves of DDC and SYT11 in the GSE39716 cohort. [file 10528_2024_10733_MOESM7_ESM.pdf]

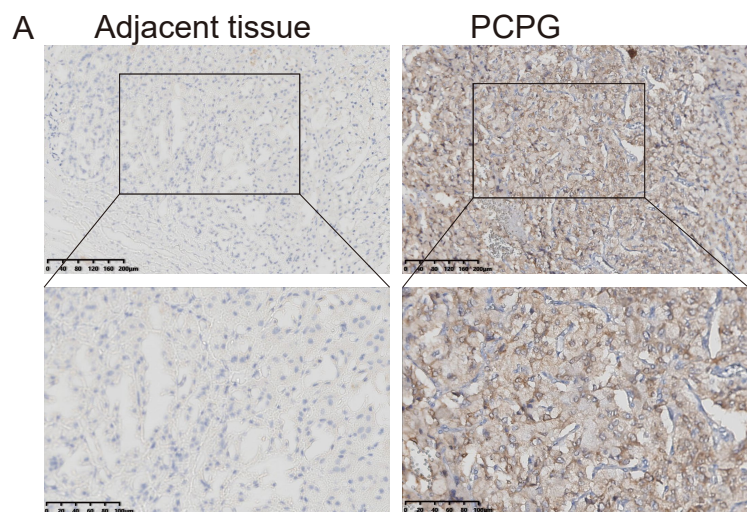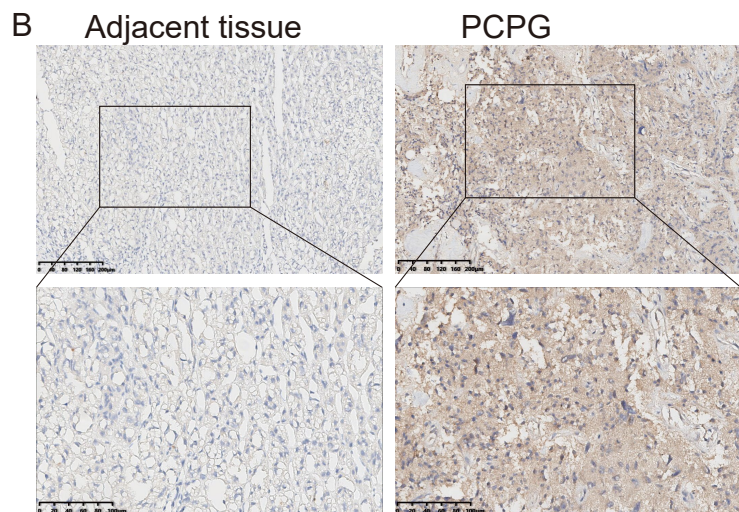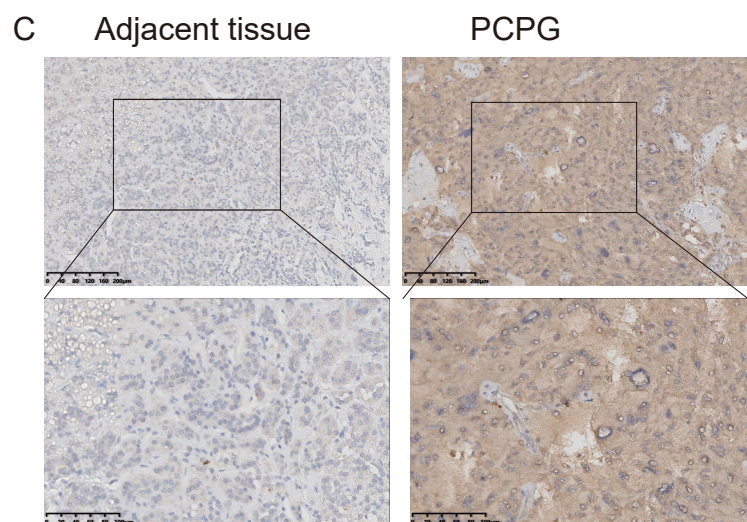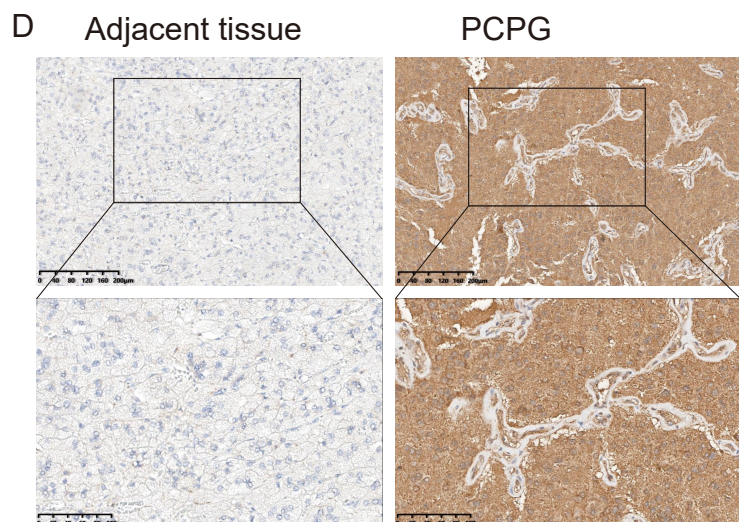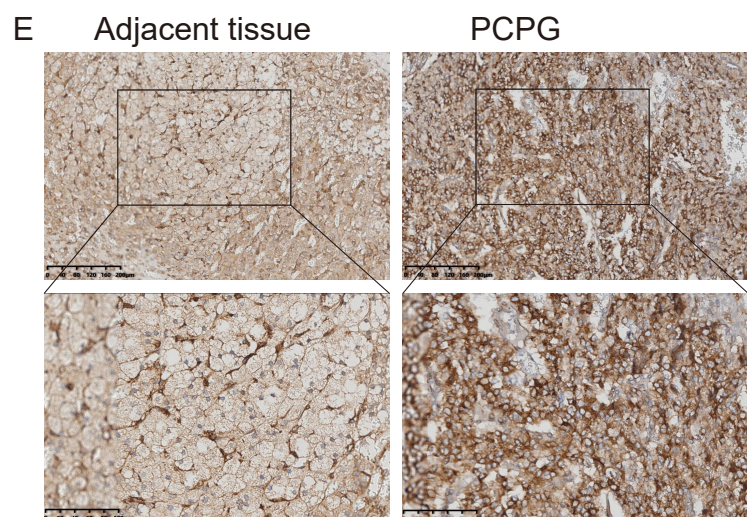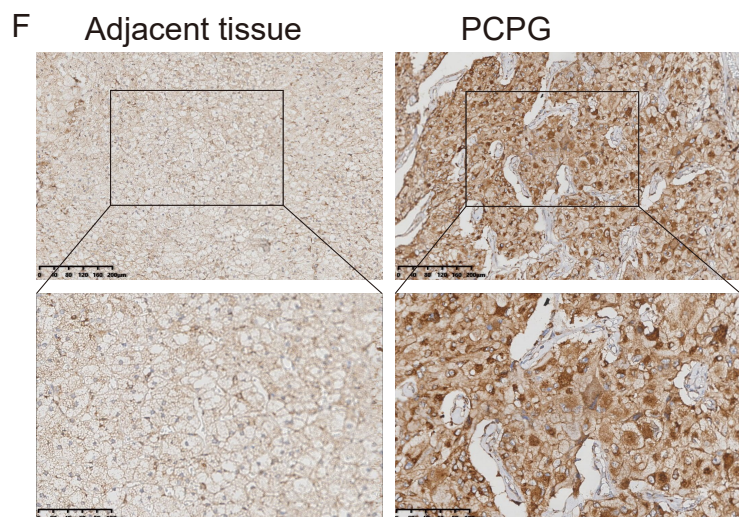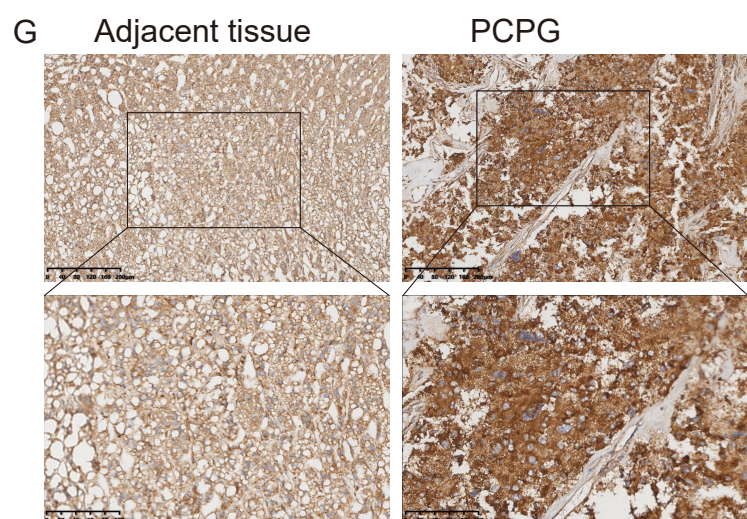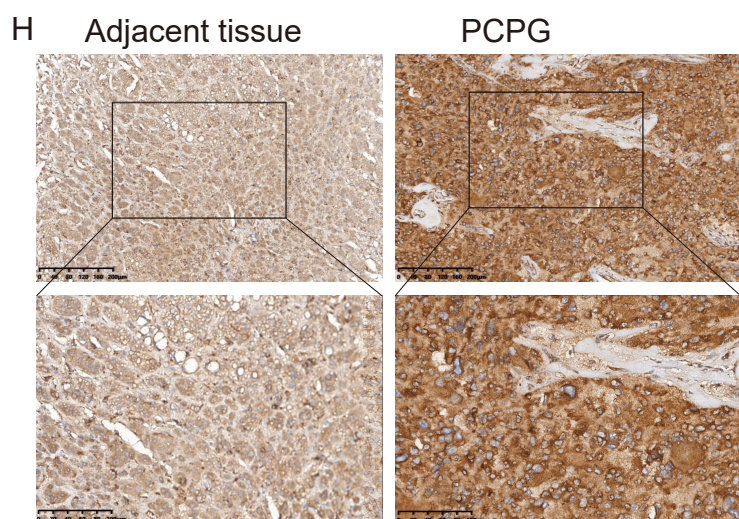

Supplement: Supplementary file 8 — Supplementary file8 (PDF 7772 kb)—Figure S4 Additional Validation of DDC and SYT11 Protein Expression Through Immunohistochemistry. (A-D) Additional representative images of IHC staining showing DDC protein expression in PCPG. (E-H) Additional representative images of IHC staining showing SYT11 protein expression in PCPG. [file 10528_2024_10733_MOESM8_ESM.pdf]
